# Preoperative Hematocrit Concentration and the Risk of Stroke in Patients Undergoing Isolated Coronary-Artery Bypass Grafting

**DOI:** 10.1155/2013/206829

**Published:** 2013-04-30

**Authors:** Khaled M. Musallam, Faek R. Jamali, Frits R. Rosendaal, Toby Richards, Donat R. Spahn, Kaivan Khavandi, Iskandar Barakat, Benjamin Demoss, Luca A. Lotta, Flora Peyvandi, Pier M. Sfeir

**Affiliations:** ^1^Department of Internal Medicine, American University of Beirut Medical Center, P.O. Box 11-0236, Beirut 1107 2020, Lebanon; ^2^Angelo Bianchi Bonomi Haemophilia and Thrombosis Center, Department of Medicine and Medical Specialties, IRCCS Ca' Granda Foundation Maggiore Policlinico Hospital, University of Milan, 20122 Milan, Italy; ^3^Division of General Surgery, Department of Surgery, American University of Beirut Medical Center, P.O. Box 11-0236, Beirut 1107 2020, Lebanon; ^4^Departments of Clinical Epidemiology and Thrombosis & Hemostasis, Leiden University Medical Center, 2333 ZA Leiden, The Netherlands; ^5^Division of Surgery and Interventional Science, University College London Hospital, London WC1E 6AU, UK; ^6^Institute of Anesthesiology, University Hospital and University of Zurich, 8006 Zurich, Switzerland; ^7^King's College London British Heart Foundation Centre, The Rayne Institute, St. Thomas' Hospital, King's Health Partners AHSC, London SE1 7EH, UK; ^8^Department of Medicine, Staten Island University Hospital, New York, NY 10305, USA; ^9^Department of Internal Medicine, Emory University School of Medicine, Atlanta, GA 30322, USA

## Abstract

*Background*. Identification and management of risk factors for stroke following isolated coronary artery bypass grafting (CABG) could potentially lower the risk of such serious morbidity. *Methods*. We retrieved data for 30-day stroke incidence and perioperative variables for patients undergoing isolated CABG and used multivariate logistic regression to assess the adjusted effect of preoperative hematocrit concentration on stroke incidence. *Results*. In 2,313 patients (mean age 65.9 years, 73.6% men), 43 (1.9%, 95% CI: 1.4–2.5) developed stroke within 30 days following CABG (74.4% within 6 days). After adjustment for a priori defined potential confounders, each 1% drop in preoperative hematocrit concentration was associated with 1.07 (95% CI: 1.01–1.13) increased odds for stroke (men, OR: 1.08, 95% CI: 1.01–1.16; women, OR: 1.02, 95% CI: 0.91–1.16). The predicted probability of stroke for descending preoperative hematocrit concentration exceeded 2% for values <37% (<37% for men (adjusted OR: 2.39, 95% CI: 1.08–5.26) and <38% for women (adjusted OR: 2.52, 95% CI: 0.53–11.98), with a steeper probability increase noted in men). The association between lower preoperative hematocrit concentration and stroke was evident irrespective of intraoperative transfusion use. *Conclusion*. Screening and management of patients with low preoperative hematocrit concentration may alter postoperative stroke risk in patients undergoing isolated CABG.

## 1. Introduction

Although mortality rates for patients undergoing isolated coronary-artery bypass grafting (CABG) continue to decline, postoperative neurologic morbidity remains a concern [1]. The short-term incidence of stroke after isolated CABG is close to 2%, although reported risks vary depending on the underlying risk factors of the population under evaluation and the adopted definition of stroke [1]. Knowledge of the mechanisms of the occurrence of stroke in patients undergoing isolated CABG is limited. It was previously assumed that strokes are attributed to the use of extracorporeal cardiopulmonary bypass. However, several studies showed similar stroke rates between patients who undergo off-pump compared with conventional on-pump surgery [2–10]. Thus, efforts to reduce the incidence of stroke now focus on identifying other perioperative and patient-related risk factors. With this background in mind, we used data from the large, multicenter database of the American College of Surgeons National Surgical Quality Improvement Program (ACS NSQIP) to determine the effects of preoperative hematocrit concentration on the incidence of stroke in patients undergoing isolated CABG.

## 2. Materials and Methods

### 2.1. Study Design and Sample

This was a cohort study using data from the ACS NSQIP database. Details of the ACS NSQIP (http://www.acsnsqip.org/) have been recently described and are summarized as follows [11].

*The American College of Surgeons National Surgical Quality Improvement Program*

*Aim. *The American College of Surgeons National Surgical Quality Improvement Program (ACS NSQIP) was set up as a rigorous data collection tool for outcome measurement. The ACS NSQIP collects data on a variety of clinical variables, including preoperative risk factors, intraoperative variables, and 30-day postoperative mortality and morbidity outcomes for patients undergoing major surgical procedures in both the inpatient and outpatient setting. Using validated statistical tools that were developed and tested in large population-based studies, the ACS NSQIP program generates an expected outcome based on the case complexity mix. The observed outcomes are then compared to the expected outcomes to obtain an O/E ratio and indicate performance of a particular medical center with regards to the national average. This data is then used to identify areas in need of quality improvement. 

*Participants. *Contribution to the ACS NSQIP is on a voluntary basis. Nonveterans hospitals that are interested in quality assurance and outcomes measurement sign-up for inclusion into the ACS NSQIP database and enter cases prospectively into this database. There are currently 289 out of 440 participating sites that are importing data to the ACS NSQIP database, located in 42 states across the US (272 sites), three Canadian provinces (15 sites), Lebanon (1 site) and the UAE (1 site). 52% of the enrolled medical centers are classified as academic/teaching and 48% are nonteaching sites. 45% of participating medical centers have ≥500 beds, 41% have 300–499 beds, 11% have 100–299 beds, and 3% have <100 beds. 

*Inclusion/Exclusion of Cases. *The ACS NSQIP includes all major surgeries as determined by CPT codes. The ACS NSQIP has developed a comprehensive Current Procedural Terminology (CPT) Code Inclusion List available on the website (http://www.acsnsqip.org/). Excluded cases are the following.

- Patients under the age of 16 years (data collected after the year 2008 was for patients over 18 years).
- Cases listed on the CPT Code Exclusion List on the website (http://www.acsnsqip.org/).
- Trauma cases—specifically: a patient who is admitted to the hospital with acute trauma and has surgery(s) for that trauma will be excluded. Any operation performed after the patient has been discharged from the trauma stay will be included.
- Transplant cases—specifically: a patient who is admitted to the hospital for a transplant and has a transplant procedure and any additional surgical procedure during the transplant hospitalization will be excluded. Any operation performed after the patient has been discharged from the transplant stay will be included.
- American Society of Anesthesiologists score 6 (brain-death organ donors).
- Concurrent case—an additional operative procedure performed by a different surgical team under the same anesthetic (e.g., coronary artery bypass graft procedure on a patient who is also undergoing a carotid endarterectomy). An assessment is not required on the concurrent procedure; however, this procedure would be reported as “concurrent” in the operative section for the assessed case.

To ensure a diverse surgical case mix, also excluded (at each center) are the following.

- More than 3 inguinal herniorrhaphies in an 8-day period.
- More than 3 breast lumpectomies in an 8-day period.
- More than 3 laparoscopic cholecystectomies in an 8-day period.
- If the site is collecting urology cases, more than 3 transurethral resection of the prostate and/or transurethral resection of bladder tumor in an 8-day period.

It is a validated outcomes registry designed to provide feedback to member hospitals on 30-day risk-adjusted surgical mortality and morbidity [12, 13]. The database includes deidentified data on demographics, preoperative risk factors and laboratory tests, intraoperative conditions and occurrences, and 30-day postoperative outcomes for adult patients undergoing major surgery in participating nonveteran's administration hospitals [12]. Trained surgical clinical reviewers collect patient data upon admission from the medical chart, operative log, anesthesia record, interviews with the surgical attending, and telephone interviews with the patient [12]. Data quality is ensured through comprehensive training of the nurse reviewers, an interrater reliability audit of participating sites, regular conference calls, and an annual meeting [14].

For this study, the available ACS NSQIP participant use files of the years 2008 (271,368 patients from 211 sites) and 2009 (336,190 patients from 237 sites) were retrieved for major surgeries performed within various surgical subspecialties at participating ACS NSQIP medical centers in the US, Canada, Lebanon, and UAE. We identified all isolated primary CABG cases using the Current Procedural Terminology (CPT) codes: 33510–33514, 33516–33519, 33521–33523, 33530, and 33533–33536. A total of 2313 patients were identified and included in this study. In accordance with the American University of Beirut's guidelines (which follow the US Code of Federal Regulations for the Protection of Human Subjects), institutional review board approval was not needed or sought for our analysis because data were collected as part of a quality assurance activity. 

### 2.2. Stroke

The ACS NSQIP registers data on stroke occurrence within 30-days of the index operation, which is defined as a focal brain dysfunction lasting ≥24 hours from a vascular cause. This definition of stroke encompasses intracranial hemorrhage, but we considered this outcome as a surrogate of ischemic stroke because hemorrhagic strokes make up only 1% of perioperative strokes [15, 16].

### 2.3. Preoperative Hematocrit Concentration

Retrieved preoperative hematocrit concentration reflected the last hematocrit measurement prior to the index operation. Some 99.9% of the hematocrit levels were obtained within eight weeks of the index surgery, 99.1% were obtained within four weeks and 96.6% were obtained within two weeks. 

### 2.4. Statistical Analysis

Descriptive statistics are presented as means (standard deviation (SD)), medians (interquartile range (IQR)), or percentages. The primary study outcome measure was stroke within 30 days of surgery. We used multivariate logistic regression analysis to retrieve effect estimates (odds ratios (OR) and 95% confidence intervals (CI)) upon adjusting the association between preoperative hematocrit concentration and the outcome of stroke for potential confounders. Models were built by adjusting (Enter method) the determinant variable (preoperative hematocrit concentration) to a priori defined potential confounders of clinical relevance (risk factors that may cause both preoperative hematocrit concentration alterations as well as stroke). Two levels of adjustment were used, Model 1 (OR_adj-1_) with basic adjustment for the most clinically relevant variables and Model 2 (OR_adj-2_) with extended adjustment for a larger number of clinically relevant risk factors. Data were near complete, with the exception of missing values for preoperative hematocrit concentration (*n* = 18, 0.7%) and body mass index (*n* = 42, 1.8%) which were imputed by the respective means of similar sex and age groups.

We carried out the data management and analyses using the SAS software version 9.1 (SAS Institute Inc., Cary, NC, USA).

## 3. Results

**Figure 1:**
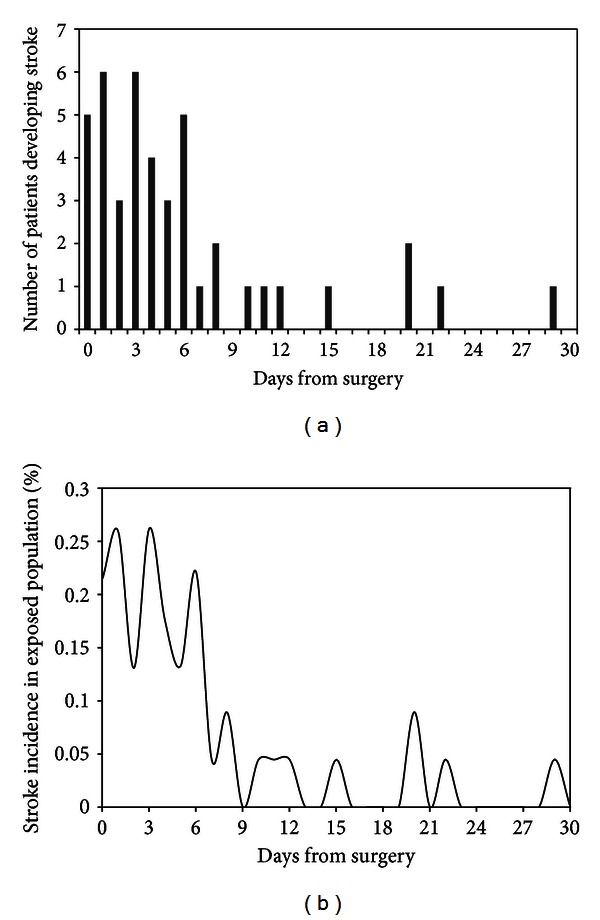
(a) Number of patients developing stroke on each day in the 30-day observation period following surgery. (b) Instantaneous stroke incidence per day in the exposed population.

A total of 2,313 patients undergoing isolated CABG were included in this analysis. The mean age of the study cohort was 65.9 years (SD: 10.7, range: 25–90) with 1,703 (73.6%) patients being men. The mean preoperative hematocrit concentration was 38.8% (SD: 5.1, range: 12.4–54.9). Forty-three patients developed stroke within 30 days following CABG, corresponding to a 30-day cumulative incidence of 1.9% (95% CI: 1.4–2.5). The median time to development of stroke was 4 days (IQR: 1–7 days, min: same day, max: 29 days), with most patients (74.4%, 95% CI: 59.7–85.0) developing stroke within the first 6 days after surgery (Figure 1). Characteristics of patients who developed and those who did not develop stroke are summarized in Table 1. Patients who developed stroke had a lower mean preoperative hematocrit concentration than those who did not (36.3% versus 38.8%, mean difference: 2.6%, 95% CI: 1.1–4.1).

In an unadjusted analysis, each drop of 1% in preoperative hematocrit concentration (continuous variable) was associated with a 1.09 increased odds of 30-day postoperative stroke (95% CI: 1.04–1.15). The effect was steeper and more certain in men (OR: 1.11, 95% CI: 1.04–1.18) than women (OR: 1.05, 95% CI: 0.93–1.17). After adjustment for potential confounders, the effect estimates dropped minimally (OR_adj-2_ 1.07, 95% CI: 1.01–1.13; men, OR_adj-2_: 1.08, 95% CI: 1.01–1.16; women, OR_adj-2_: 1.02, 95% CI: 0.91–1.16) (Table 2). 

**Figure 2:**
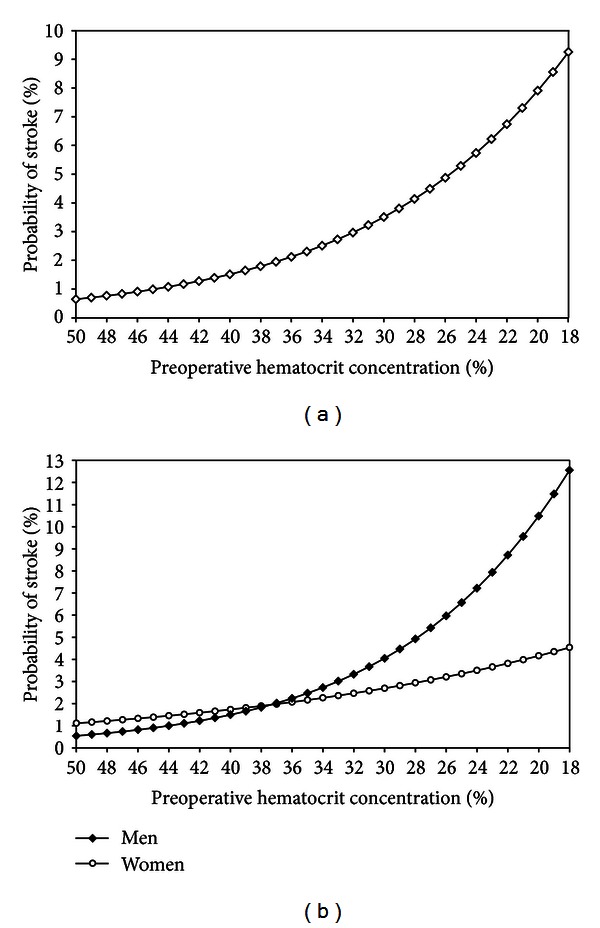
Predicted probability of 30-day postoperative stroke as a function of preoperative hematocrit concentration in (a) all patients and (b) men and women.

The predicted probability of stroke for descending preoperative hematocrit concentration values in a clinically relevant range is illustrated in Figure 2(a). The predicted probability of stroke exceeded 2% (cumulative incidence in this study and incidence commonly reported in previous studies) for preoperative hematocrit concentration values <37% (Figure 2(a)). Upon stratification for men and women, the threshold was <37% for men and <38% for women, with a steeper probability increase noted in men beyond the threshold (Figure 2(b)). The adjusted increase in odds of stroke (OR_adj-2_) for men with a preoperative hematocrit concentration <37% compared with ≥37% was 2.39 (95% CI: 1.08–5.26), while the adjusted increase in odds of stroke (OR_adj-2_) for women with a preoperative hematocrit concentration <38% compared with ≥38% was 2.52 (95% CI: 0.53–11.98) (Table 2).

**Figure 3:**
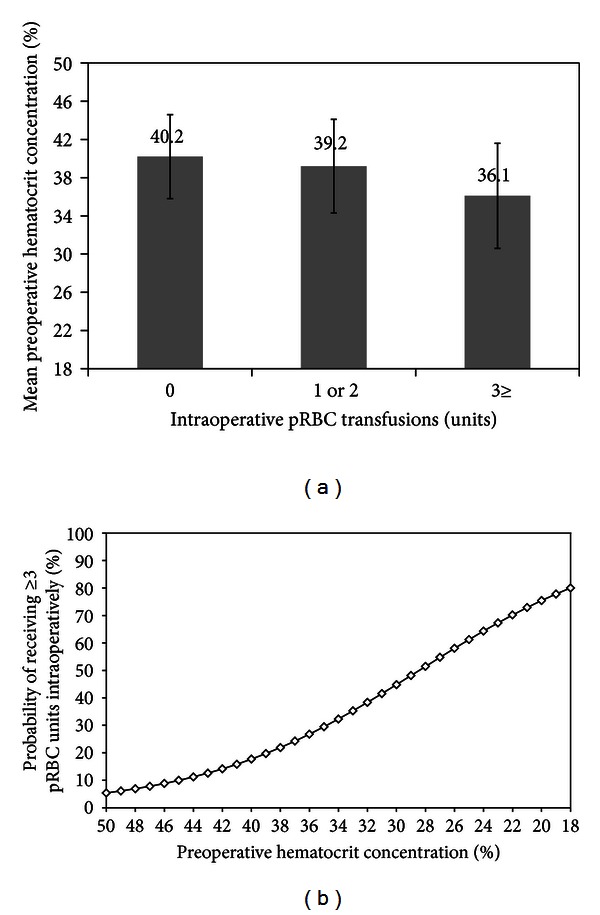
(a) Bar chart showing mean preoperative hematocrit concentration according to the number of pRBC units transfused intraoperatively, whiskers present standard deviation. (b) Predicted probability of receiving 3 or more pRBC units intraoperatively as a function of preoperative hematocrit concentration. pRBC = packed red blood cell.

A total of 1,779 (76.9%, 95% CI: 75.2–78.6) patients received intraoperative transfusions (55.1% received 1 or 2 packed red blood cell (pRBC) units and 21.8% received 3 or more pRBC units). The odds of 30-day stroke were more notably increased in patients receiving 3 or more pRBC units (OR: 2.69, 95% CI: 1.04–7.00) than patients receiving 1 or 2 pRBC units (OR: 1.55, 95% CI: 0.62–3.84) when compared to patients who did not receive intraoperative transfusions. The mean preoperative hematocrit concentration was lower in patients who received 3 or more pRBC units intraoperatively than those who did not (36.1% versus 39.5%, mean difference: 3.4%, 95% CI: 2.9–3.9) (Figure 3(a)). The odds of receiving 3 or more pRBC intraoperatively were 1.14 (95% CI: 1.12–1.17) for each 1% drop in preoperative hematocrit concentration (Figure 3(b)). 

The association between preoperative hematocrit concentration (continuous variable) and 30-day postoperative stroke was observed in both patients who received (OR_adj-2_: 1.10, 95% CI: 1.02–1.18) and those who did not receive (OR_adj-2_: 1.04, 95% CI: 1.01–1.08) 3 or more pRBC units intraoperatively, although with higher effect noted in patients who received intraoperative transfusions. This indicated that the effects of preoperative hematocrit concentration on 30-day postoperative stroke are not solely mediated by the use of 3 or more intraoperative pRBC transfusions.

## 4. Discussion

The incidence of stroke in our study, relying on data from isolated CABG procedures performed in 2008 and 2009, was 1.9%, which is in close agreement to recent reports [17]. It should be noted, however, that estimated rates are much higher when asymptomatic infarcts are included in the definition of stroke. In addition, strokes may be missed in the perioperative period, since patients are often receiving pain medications or sedatives, which can mask subtle neurologic signs [1]. The study by Tarakji and colleagues reported an intraoperative occurrence in 40% of overall stroke cases (a total of 705 patients developed stroke among 45432 participants in the study) [17]. None of the 43 cases in this report had stroke documentation intraoperatively, although 5 (11.6%) developed stroke on the same day of surgery. However, the distribution of stroke risk per day in the postoperative period in our study matches that reported by Tarakji and colleagues [17], with a peak incidence during the first 6 days following surgery followed by a decline in risk. 

We identified an association between descending preoperative hematocrit concentration values and an increased risk of stroke in the 30-day period following isolated CABG. The increased risk of stroke exceeded 2% in patients with a preoperative hematocrit concentration <37%. Moreover, the increased postoperative stroke risk attributed to declining preoperative hematocrit concentration values was more notable in men than women and was independent of yet augmented by the excessive use of intraoperative pRBC transfusions.

Although numerous studies identified the effects of perioperative hematocrit alterations and pRBC transfusions on morbidity and mortality in cardiac surgery, very few reports evaluated the outcome of stroke in specific. An association between hemodilutional anemia during cardiopulmonary bypass (nadir intraoperative hematocrit levels) and the incidence of stroke was previously demonstrated [18, 19]. One study by Kulier and colleagues showed that both low preoperative hemoglobin (<11.0 g/dl) and intraoperative transfusion requirement are predictors of postoperative adverse noncardiac outcomes (a composite end point of neurological and renal events) in patients undergoing CABG [20]. A more recent study showed that preoperative hemoglobin <12.5 g/dl in both men and women is associated with increased odds of the composite outcome of death, stroke, or acute kidney injury in cardiac surgery patients, irrespective of intraoperative pRBC transfusion use; however, there was no association with the outcome of stroke alone [21]. In this study, we echo these findings and confirm the independent contribution of low preoperative hematocrit concentration on stroke incidence, although a more prominent effect is observed in patients who require intraoperative transfusions of 3 or more pRBC units.

Previous studies suggest that intraoperative hypotension and subsequent hypoperfusion may be a source of neurologic injury in patients undergoing CABG [1, 22, 23]. Preoperative low hematocrit concentration and significant intraoperative blood loss necessitating excessive transfusion [24, 25] may both lead to a state of cerebral hypoperfusion; which in the coexistence of other risk factors (vessel atherosclerosis) may lead to adverse cerebral events. Of note, patients with chronic hypertension may be exposed to relative intraoperative hypotension to the brain if their blood pressure is maintained at a normal or slightly low level during surgery, thus placing them at risk for a watershed stroke [26, 27]. The use of intraoperative transfusions also leads to immunemodulation. Transfusions may lead to immune activation and induce multiple organ failure, despite leukoreduction [28]. In fact, systemic inflammation has been associated with neurologic injury in patients undergoing CABG [1, 22]. Moreover, storage-related posttransfusion hemolysis producing hemoglobin-driven pathophysiology and thrombogenic RBC microparticles in stored blood can lead to target organ damage [29–32]. Thus, our findings support recommendations of a conservative approach to intraoperative blood use in patients undergoing isolated CABG [33] and should lead to a careful consideration of appropriate interventions aimed at correcting preoperatively low hematocrit levels.

Our study carries several limitations. The ACS NSQIP database does not record intraoperative nadir hematocrit or immediate postoperative hematocrit. Thus, we could not evaluate the association between these variables and a stroke outcome. Moreover, the database does not record data on cardiovascular drug use. The ACS NSQIP database also does not document the means by which stroke was diagnosed. The use of magnetic resonance imaging rather than computed tomography may result in higher rates of radiographic infarct. Although the types of imaging techniques used may have affected the observed incidence of stroke in our study, an association between the use of a certain imaging technique and the evaluated risk factors is less likely. Another potential limitation of this study was that we were unable to control for hospital effects owing to the absence of hospital identifiers in our data. There may have been variability in hospital quality or variability in surgical strategy which may have potentially confounded the association between risk factors and outcome. Finally, the possibility of residual confounding is always present in observational studies.

## 5. Conclusions

Current efforts continue to focus on reducing the embolic burden during CABG, being considered as the primary mechanism through which neurologic injury occurs. Our study shows that other mechanisms of injury could be involved. An increasing number of surgical centers now use preoperative screening to identify patients who have an increased risk for stroke, and to modify surgical conditions according to the results of such screening. This approach should ideally become standard of care.

## Figures and Tables

**Table 1:** Patients' characteristics.

Parameter	No stroke *n* = 2270	Stroke *n* = 43
Preoperative hematocrit concentration, mean (SD)	**38.8 (5.1)**	**36.3 (4.9)**
Age in years, mean (SD)	65.9 (10.7)	69.3 (9.2)
Male, *n* (%)	1,673 (73.7)	30 (69.8)
White race, *n* (%)	1,866 (82.2)	37 (86.0)
Body mass index ≥ 30 kg/m^2^, *n* (%)	974 (42.9)	15 (34.9)
Diabetes, *n* (%)	831 (36.6)	19 (44.2)
Hypertension, *n* (%)	1,914 (84.3)	39 (90.7)
Congestive heart failure, *n* (%)	255 (11.2)	8 (18.6)
Peripheral vascular disease, *n* (%)	119 (5.2)	2 (4.7)
Currently on dialysis, *n* (%)	56 (2.5)	4 (9.3)
Current Smoker, *n* (%)	554 (24.2)	15 (34.9)
Chronic obstructive pulmonary disease, *n* (%)	237 (10.4)	9 (20.9)
History of transient ischemic attack, *n* (%)	138 (6.1)	4 (9.3)
History of stroke with neurologic deficit, *n* (%)	100 (4.4)	4 (9.3)
History of stroke without neurologic deficit, *n* (%)	91 (4.0)	1 (2.3)
Bleeding disorder, *n* (%)	366 (16.1)	8 (18.6)
Disseminated cancer, *n* (%)	3 (0.1)	0 (0.0)
Tumor involving central nervous system, *n* (%)	1 (0.0)	0 (0.0)

**Table 2:** Effects of preoperative hematocrit concentration on 30-day postoperative stroke.

Variable	Odds of stroke		
	OR_unadj_ (95% CI)	OR_adj-1_ (95% CI)	OR_adj-2_ (95% CI)
Preoperative hematocrit concentration (continuous variable, per 1% decrease)			
All patients (*n* = 2,313)	1.09 (1.04–1.15)	1.09 (1.03–1.15)	1.07 (1.01–1.13)
Men (*n* = 1,703)	1.11 (1.04–1.18)	1.10 (1.04–1.17)	1.08 (1.01–1.16)
Women (*n* = 610)	1.05 (0.93–1.17)	1.04 (0.92–1.17)	1.02 (0.91–1.16)
Preoperative hematocrit concentration (categorized)			
All patients <37% versus ≥37%	1.92 (1.05–3.51)	1.76 (0.93–3.35)	1.49 (0.76–2.91)
Men <37% versus ≥37%	3.07 (1.49–6.34)	2.80 (1.34–5.88)	2.39 (1.08–5.26)
Women <38% versus ≥38%	2.88 (0.63–13.10)	2.71 (0.59–12.42)	2.52 (0.53–11.98)

OR_unadj_: Unadjusted odds ratio.

OR_adj-1_: Adjusted odds ratio according to Model 1. Adjusted for age, sex, and race.

OR_adj-2_: Adjusted odds ratio according to Model 2. Adjusted for all variables in Table 1.
